# Simplified acute physiology score III is excellent for predicting in-hospital mortality in coronary care unit patients with acute myocardial infarction: A retrospective study

**DOI:** 10.3389/fcvm.2022.989561

**Published:** 2022-12-08

**Authors:** Xiaoyu Zheng, Tianyang Hu, Tingrong Liu, Wei Wang

**Affiliations:** ^1^School of Clinical Medicine, Chongqing Medical and Pharmaceutical College, Chongqing, China; ^2^Precision Medicine Center, The Second Affiliated Hospital, Chongqing Medical University, Chongqing, China; ^3^Department of Geriatrics, The People’s Hospital of Yubei District of Chongqing City, Chongqing, China; ^4^Department of Orthopedics, The People’s Hospital of Yubei District of Chongqing City, Chongqing, China

**Keywords:** SAPS III, SAPS II, OASIS, LODS, acute myocardial infarction, in-hospital mortality, coronary care unit

## Abstract

**Background:**

Coronary care unit (CCU) patients with acute myocardial infarction (AMI) lack effective predictors of in-hospital mortality. This study aimed to investigate the performance of four scoring systems in predicting in-hospital mortality in CCU patients with AMI.

**Methods:**

The baseline data, the logistic organ dysfunction system (LODS), the Oxford acute severity of illness score (OASIS), the simplified acute physiology score II (SAPS II), and the simplified acute physiology score III (SAPS III) scores of the patients were extracted from the fourth edition of the Medical Information Mart for Critical Care (MIMIC-IV) database. Independent risk factors for in-hospital mortality were identified by regression analysis. We performed receiver operating characteristic (ROC) curves and compared the area under the curve (AUC) to clarify the predictive value of the four scoring systems. Meanwhile, Kaplan–Meier curves and decision curve analysis (DCA) were performed to determine the optimal scoring system for predicting in-hospital mortality.

**Results:**

A total of 1,098 patients were included. The SAPS III was an independent risk factor for predicting in-hospital mortality in CCU patients with AMI before and after the propensity score matching (PSM) analysis. The discrimination of in-hospital mortality by SAPS III was superior to that of LODS, OASIS, and SAPS II. The AUC of the SAPS III scoring system was the highest among the four scoring systems, at 0.901 (before PSM) and 0.736 (after PSM). Survival analysis showed that significantly more in-hospital mortality occurred in the high-score SAPS III group compared to the low-score SAPS III group before PSM (HR 7.636, *P* < 0.001) and after PSM (HR 2.077, *P* = 0.005). The DCA curve of SAPS III had the greatest benefit score across the largest threshold range compared to the other three scoring systems.

**Conclusion:**

The SAPS III was an independent risk factor for predicting in-hospital mortality in CCU patients with AMI. The predictive value for in-hospital mortality with SAPS III is superior to that of LODS, OASIS, and SAPS II. The results of the DCA analysis suggest that SAPS III may provide a better clinical benefit for patients. We demonstrated that SAPS III is an excellent scoring system for predicting in-hospital mortality for CCU patients with AMI.

## Introduction

As a common type of coronary heart disease (CHD), AMI is a fatal and seriously life-threatening disease. According to some reports, the in-hospital mortality rate for ST-elevation myocardial infarction (STEMI) is approximately 10% (1–3). The mortality rate for patients with AMI treated in a regular ward dropped from 26 to 7% for those treated in the CCU because of specialized care from the CCU (4). Predicting in-hospital mortality in CCU patients with AMI could help improve the treatment and prognosis of AMI-related complications. Although CCU treatment benefits patients, it lacks an efficient and practical scoring system to predict in-hospital mortality.

Nowadays, many risk-scoring tools are used to assess mortality from AMI (5–7). They may provide some predictive value for patients with AMI. However, they have limitations, such as insufficient clinical data for developing risk scores, low predictive efficiency, and unaccepted novel scoring tools that hinder their widespread use. Therefore, it is vital to identify an acceptable and effective scoring method for predicting in-hospital mortality in CCU patients with AMI.

Various scoring systems in intensive care have demonstrated outstanding performance in predicting in-hospital mortality. As a generally acceptable scoring system, the logistic organ dysfunction system (LODS) can be used to predict morbidity and mortality in intensive care unit (ICU) patients and quantify their baseline severity of organ dysfunction (8, 9). The Oxford acute severity of illness score (OASIS) contains minimal variables for assessing disease mortality in the ICU. These machine-learning algorithms guarantee the accuracy of predictions (10). The SAPS II and SAPS III are developed to provide estimates of ICU admission mortality (11, 12). Compared to SAPS II, SAPS III has a higher-quality multinational database for predicting mortality before ICU intervention (13). However, in an ICU for internal disorders, SAPS II seemed to show better mortality prediction performance than SAPS III (14). Our study aimed to explore the utility of the above scoring systems in evaluating the in-hospital mortality of CCU patients admitted for AMI and to investigate the better performance of the prediction models in clinical practice.

## Materials and methods

### Database

This retrospective study included hospital admissions from the MIMIC-IV database. All real hospital stays of patients between 2008 and 2019 admitted to the critical care units of the Beth Israel Deaconess Medical Center (Boston, MA, USA) were included in the study. Regarding introductions to the database, refer to the official website^1^ : Detailed data from MIMIC-IV (version 1.0) were obtained through PhysioNet.^2^ Author XZ passed the “Protecting Human Research Participants” examination on the National Institutes of Health website and signed a data usage agreement (Record ID: 48747466) to access the database. The patient’s private information (real name, family address, and telephone) in the database is anonymous. Therefore, ethical approval and informed consent are not required. This study complies with the Declaration of Helsinki.

### Study population and data extraction

Data were extracted by Navicat Premium software (version 15.0). All CCU patients diagnosed with AMI were screened, and the following data were extracted from the MIMIC-IV database: age, gender, length of hospital stay, and length of CCU stay. The Charlson comorbidity index (CCI) (15) provided a simple and effective method for assessing comorbidities. Coexisting comorbidities, such as congestive heart failure (CHF), cerebrovascular disease (CVD), chronic pulmonary disease (CPD), diabetes, hypertension, renal disease, liver disease, and malignant cancer (MC), were included. We also investigated whether patients had a combined acute kidney injury (AKI) during their hospitalization. Laboratory results (hemoglobin, white blood cell, platelets, anion gap, blood urea nitrogen, serum creatinine, and the international normalized ratio), vital signs (heart rate, mean blood pressure, respiratory rate, and temperature), mechanical ventilation (MV), and scoring systems (LODS, OASIS, SAPS II, and SAPS III) should be recorded within 24 h of admission. If variables related to laboratory results or vital signs were assessed multiple times, then the average value was taken. We investigated whether patients underwent percutaneous coronary intervention (PCI) or coronary artery bypass grafting (CABG) during hospitalization. Only patients admitted to the ICU for the first time were included.

### Statistical analysis

Normally distributed continuous variables (Kolmogorov–Smirnov tests evaluated normality) were expressed as mean ± standard deviation, and the independent samples *t*-test was used for comparison. Non-normally distributed continuous variables were expressed as the median of the interquartile range (IQR), and the Wilcoxon rank-sum test was applied to the comparison. Categorical variables were expressed as percentages and compared using the chi-square test. Independent risk factors for in-hospital mortality in CCU patients with AMI were determined by binomial logistic regression and Cox regression. In the multivariate analysis, we only considered the variables that had a P-value < 0.1 in the univariate analysis. The ROC curves of the four scoring systems for predicting in-hospital mortality were drawn, and the area under the curve (AUC) was compared using the Delong method (16) to determine the discriminative power of each scoring system.

To reduce baseline bias, propensity score matching (PSM) analysis was used to correct some confounding factors, making comparisons between the death and survival groups more reasonable. A 1:1 nearest neighbor matching algorithm (caliper = 0.05, without replacement) conducted the PSM analysis, and a logistic regression model calculated the propensity score. The following variables were included: age, gender, CCI, LODS, OASIS, SAPS II, SAPS III, AG, MV, PCI/CABG, and AKI. In addition, once a better scoring system was determined, Kaplan–Meier survival curves were drawn using the optimal cutoff value of the ROC curve to further clarify the value of the scoring system in predicting in-hospital mortality. The log-rank test was utilized to identify any differences between the groups. The decision curve analysis (DCA) was performed to evaluate the net benefits of the four scoring systems in CCU patients with AMI after PSM (17). The above statistical analyses were performed using R (version 4.1.2) software and MedCalc software (version 20.1.0). A *P*-value <0.05 was defined as statistically significant.

## Results

### Baseline characteristics

**FIGURE 1:**
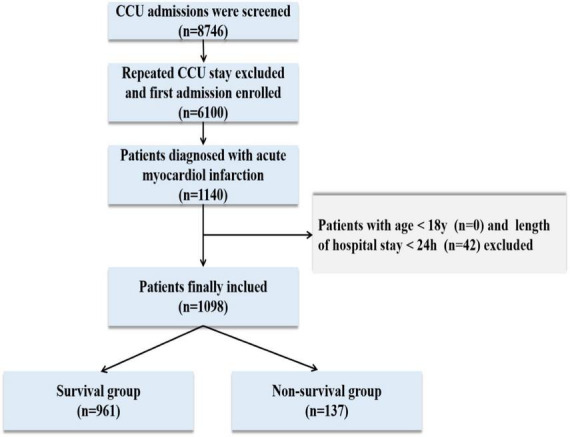
Flowchart of the study cohort. CCU, coronary care unit.

A total of 76,540 ICU admissions were included in the MIMIC-IV database, including 8,746 CCU patients. Finally, 1,098 patients were carefully screened, of whom 137 died and 961 survived in the hospital. The data selection flowchart is shown in Figure 1. The median age of the death group was 76 years, which was older than the median age of the survival group of 69 years (*P* < 0.001). The patients who died had a longer length of CCU stay when compared with the patients who survived. On the first day of admission, CCI, white blood cell, blood urea nitrogen, serum creatinine, anion gap, international normalized ratio, heart rate, respiratory rate, LODS score, OASIS score, SAPS II score, and SAPS III scores in the death group were significantly greater than those in the survival group (*P* < 0.001 for all). A lower Hb level, PLT, and MAP were found in the death group than in the survival group (*P* < 0.001 for all). Moreover, non-survivors were more likely than survivors to have comorbidities, such as CHF, CVD, diabetes, liver disease, renal disease, and AKI (*P* < 0.05 for all). The proportion of patients who received MV was remarkably higher in the death group (*P* < 0.001), while there was no statistical difference in the length of hospital stay and whether patients underwent PCI/CABG. PSM balanced variables including age, gender, length of hospital stay, length of CCU stay, whether patients underwent PCI/CABG, mechanical ventilation, AKI, and CCI. After matching, the two groups of variables were comparable (*P* > 0.05 for all), and 108 dead patients and 108 surviving patients were finally included. After PSM, the death group had higher levels of BUN, Cr, INR, and AG than the surviving group. The differences among the four scoring systems were similar to those before matching (*P* < 0.05 for all). There was no statistical difference in coexisting comorbidities between the two groups, except for minor differences in CPD. Baseline characteristics and detailed data are shown in Table 1.

**TABLE 1:** Demographic and clinical characteristics of patients.

	Before PSM			After PSM		
Characteristics	Death (*n* = 137)	Survival (*n* = 961)	*P*	Death (*n* = 108)	Survival (*n* = 108)	*P*
Age, year	76.0 (67.5, 85.5)	69.0 (59.0, 79.0)	<0.001	75.0 (67.0, 85.0)	77.0 (68.3, 83.8)	0.642
Gender (male)	77 (56.2%)	619 (64.4%)	0.062	61 (56.5%)	55 (50.9%)	0.413
LOS Hos, day	4.9 (2.3, 9.7)	4.3 (3.0, 7.9)	0.418	5.09 (2.51, 9.20)	6.9 (4.0, 10.6)	0.001
LOS CCU, day	3.5 (1.6, 7.0)	1.7 (1.0, 3.0)	<0.001	3.3 (1.5, 6.6)	3.8 (2.0, 6.8)	0.321
CCI	7.5 (6.0, 9.0)	6.0 (4.0, 8.0)	<0.001	6.0 (7.0, 8.0)	7.0 (6.0, 9.0)	0.362
**Coexisting comorbidities**						
CHF	92 (67.2%)	467 (48.6%)	<0.001	71(65.7%)	72 (66.7%)	0.886
CVD	23 (16.8%)	77 (8.0%)	0.001	16 (14.8%)	15 (13.9%)	0.846
CPD	29 (21.2%)	176 (18.3%)	0.423	17 (15.7%)	29 (26.9%)	0.046
Diabetes	60 (43.8%)	307 (31.9%)	0.006	43 (39.8%)	51 (47.2%)	0.272
Hypertension	40 (29.2%)	409 (42.6%)	0.003	32 (29.6%)	41 (38.0%)	0.195
Liver disease	13 (9.5%)	34 (3.5%)	0.001	10 (9.3%)	10 (9.3%)	1.000
Renal disease	47 (34.3%)	184 (19.1%)	<0.001	35(32.4%)	33 (3.06%)	0.770
MC	6 (4.4%)	31 (3.2%)	0.484	5 (4.6%)	7 (6.5%)	0.552
AKI	126 (92.0%)	434 (45.2%)	<0.001	97(89.8%)	94 (87.0%)	0.523
**Laboratory tests**						
Hb, g/dl	11.1 (9.6, 12.5)	12.4 (11.0, 13.9)	<0.001	11.1 (9.6, 12.5)	11.7 (10.0, 13.1)	0.365
WBC, 10^9^/L	14.2 (10.3, 17.3)	10.9 (8.6, 13.6)	<0.001	14.5 (10.5, 17.5)	13.0 (10.0, 16.8)	0.115
PLT, 10^9^/L	198 (153, 255)	215 (176, 265)	0.047	205 (161, 252)	216 (178, 261)	0.292
BUN, mmol/L	33.0 (24.8, 48.8)	18.5 (14.5, 25.0)	<0.001	33.0 (22.3, 51.3)	24.0 (17.6, 41.1)	0.002
Cr, mg/dl	1.6 (1.3, 2.3)	1.0 (0.8, 1.3)	<0.001	1.8 (1.3, 2.4)	1.2 (0.95, 1.85)	<0.001
INR	1.3 (1.2, 1.6)	1.2 (1.1, 1.3)	<0.001	1.3 (1.5, 1.6)	1.2 (1.1, 1.3)	0.003
AG, mmol/L	18.3 (15.5, 20.5)	15.0 (13.0, 17.0)	<0.001	18.5 (15.8, 21.0)	15.5 (13.5, 17.5)	<0.001
**Vital signs**						
HR (bpm)	83 (72, 97)	76 (68, 85)	<0.001	83(73, 97)	79 (69, 88)	0.013
MAP (mmHg)	76 (69, 82)	80 (73, 87)	<0.001	77(70, 81)	76 (71, 83)	0.085
RR (cpm)	21 (19, 24)	19 (17, 21)	<0.001	21(19, 24)	19 (17, 21)	<0.001
T (°C)	36.7 (36.4, 37.1)	36.8 (36.6, 37.0)	0.030	36.6 (36.5, 36.9)	36.8 (36.5, 37.1)	0.031
**Scoring systems**						
LODS	8.5 (6, 12)	2 (1, 4)	<0.001	8.0 (5.0, 11.0)	5.0 (3.0, 8.0)	<0.001
OASIS	41.5 (35, 48)	26 (22, 32)	<0.001	39.5 (33, 47)	36.0 (27.0, 44.7)	0.021
SAPS II	49 (41, 61)	29 (23, 36)	<0.001	48(40, 59)	40.0 (32.0, 50.0)	<0.001
SAPS III	70.5 (54, 97)	33(24, 44)	<0.001	69(53.5, 93)	38.5 (32.3, 64.0)	<0.001
MV	80 (58.4%)	143 (14.9%)	<0.001	53 (49.0%)	60 (55.6%)	0.340
PCI/CABG	41 (29.9%)	327 (34.0%)	0.342	36 (33.3%)	32 (29.6%)	0.558

PSM, propensity score matching; LOS, length of stay; Hos, hospital; CCU, coronary care unit; CCI, charlson comorbidity index; CHF, congestive heart failure; CVD, cerebrovascular disease; CPD, chronic pulmonary disease; MC, malignant cancer; AKI, acute kidney injury; Hb, hemoglobin; WBC, white blood cell; PLT, platelet; BUN, blood urea nitrogen; Cr, creatinine; INR, international normalized ratio; AG, anion gap; HR, heart rate; MAP, mean arterial pressure; RR, respiratory rate; T, temperature; GCS, glasgow coma scale; LODS, logistic organ dysfunction system; OASIS, oxford acute severity of illness score; SAPS, simplified acute physiology score; MV, mechanical ventilation; PCI, percutaneous coronary intervention; CABG, coronary artery bypass grafting.

### Regression analysis

#### Logistic regression analysis

Before PSM, age, AG, and SAPS III were independent risk factors for in-hospital mortality in CCU patients with AMI, regardless of whether the regression was univariable or multivariable (*P* < 0.01 for all) (Table 2). After PSM, AG (univariable regression analysis: OR 1.276, 95% CI 1.166–1.395, *P* < 0.001; multivariable regression analysis: OR 1.188, 95% CI 1.082–1.304, *P* < 0.01), OASIS (univariable regression analysis: OR 1.036, 95% CI 1.007–1.065, *P* < 0.001; multivariable regression analysis: OR 0.947, 95% CI 0.899–0.998, *P* = 0.043), and SAPS III (univariable regression analysis: OR 1.036, 95% CI 1.023–1.049, *P* < 0.001; multivariable regression analysis: OR 1.046, 95% CI 1.023–1.070, *P* = 0.001) were independent risk factors for in-hospital mortality in the included patients (Table 3).

**TABLE 2:** Binomial logistic regression analysis for in-hospital mortality among coronary care unit patients with AMI (before PSM).

Variable	Univariable		Multivariable	
	OR (95% CI)	*P*	OR (95% CI)	*P*
Age	1.041(1.026–1.056)	<0.001	1.036(1.010–1.062)	0.006
Gender (male)	0.709(0.493–1.019)	0.709		
CCI	1.284(1.194–1.381)	<0.001	1.045(0.935–1.169)	0.437
LODS	1.519(1.430–1.614)	<0.001	1.070(0.933–1.226)	0.334
OASIS	1.154(1.129–1.180)	<0.001	1.026(0.982–1.072)	0.256
SAPS II	1.108(1.091–1.125)	<0.001	1.015(0.987–1.044)	0.310
SAPS III	1.064(1.054–1.074)	<0.001	1.031(1.014–1.049)	<0.001
MV	8.028(5.472–11.78)	<0.001	0.994(0.469–2.108)	0.988
PCI/CABG	0.828(0.561–1.222)	0.342		
AKI	13.909(7.414–26.094)	<0.001	2.095(0.997–4.400)	0.051
AG	1.352(1.277–1.431)	<0.001	1.178(1.098–1.265)	<0.001

PSM, propensity score matching; AMI, acute myocardial infarction; OR, odds ratio; CI, confidence interval; CCI, charlson comorbidity index; AG, anion gap; AKI, acute kidney injury; LODS, logistic organ dysfunction system; OASIS, oxford acute severity of illness score; SAPS, simplified acute physiology score; MV, mechanical ventilation; PCI, percutaneous coronary intervention; CABG, coronary artery bypass grafting.

**TABLE 3:** Binomial logistic regression analysis for in-hospital mortality among coronary care unit patients with AMI (after PSM).

Variable	Univariable		Multivariable	
	OR (95% CI)	*P*	OR (95% CI)	*P*
Age	0.994(0.972–1.016)	0.589		
Gender (male)	1.251(0.732–2.137)	0.413		
CCI	0.942(0.834–1.065)	0.340		
LODS	1.202(1.109–1.302)	<0.001	1.029(0.863–1.225)	0.753
OASIS	1.036(1.007–1.065)	0.014	0.947(0.899–0.998)	0.043
SAPS II	1.041(1.019–1.063)	<0.001	1.002(0.966–1.039)	0.919
SAPS III	1.036(1.023–1.049)	<0.001	1.046(1.023–1.070)	0.001
MV	0.771(0.451–1.316)	0.341		
PCI/CABG	1.187(0.668–2.110)	0.558		
AKI	1.313(0.567–3.039)	0.524		
AG	1.276(1.166–1.395)	<0.001	1.188(1.082–1.304)	<0.001

PSM, propensity score matching; AMI, acute myocardial infarction; OR, odds ratio; CI, confidence interval; CCI, charlson comorbidity index; AG, anion gap; AKI, acute kidney injury; LODS, logistic organ dysfunction system; OASIS, oxford acute severity of illness score; SAPS, simplified acute physiology score; MV, mechanical ventilation; PCI, percutaneous coronary intervention; CABG, coronary artery bypass grafting.

#### Cox regression analysis

Cox regression was performed to identify the risk factors and confirm the results of binomial logistic regression. Both univariable hazards analyses and multivariate analyses revealed that SAPS III was the independent predictor of mortality in CCU patients with AMI before PSM (univariate analysis: HR 1.031, 95% CI 1.026–1.037, *P* < 0.001; multivariate analysis: HR 1.015, 95% CI 1.004–1.027, *P* = 0.006) (Table 4) and after PSM (univariate analysis: HR 1.012, 95% CI 1.005–1.019, *P* < 0.001; multivariate analysis: HR 1.014, 95% CI 1.001–1.026, *P* = 0.038) (Table 5).

**TABLE 4:** Cox regression analyses of risk factors for in-hospital mortality among coronary care unit patients with AMI (before PSM).

Variable	Univariate analysis		Multivariate analysis	
	HR (95% CI)	*P*	HR (95% CI)	*P*
Age	1.034(1.019–1.049)	<0.001	1.037(1.018–1.056)	<0.001
Gender (male)	0.787(0.561–1.103)	0.164		
CCI	1.103(1.033–1.177)	0.003	0.965(0.882–1.055)	0.431
LODS	1.250(1.200–1.301)	<0.001	0.956(0.871–1.049)	0.341
OASIS	1.088(1.071–1.105)	<0.001	1.027(0.994–1.061)	0.106
SAPS II	1.062(1.052–1.072)	<0.001	1.029(1.011–1.047)	0.002
SAPS III	1.031(1.026–1.037)	<0.001	1.015(1.004–1.027)	0.006
MV	3.204(2.262–4.539)	<0.001	0.827(0.474–1.444)	0.504
PCI/CABG	0.589(0.407–0.851)	0.005	0.630(0.428–0.927)	0.019
AKI	5.693(3.049–10.63)	<0.001	2.091(1.078–4.057)	0.029
AG	1.209(1.156–1.263)	<0.001	1.135(1.076–1.197)	<0.001

PSM, propensity score matching; AMI, acute myocardial infarction; HR, hazard ratio; CI, confidence interval; CCI, charlson comorbidity index; AG, anion gap; AKI, acute kidney injury; LODS, logistic organ dysfunction system; OASIS, oxford acute severity of illness score; SAPS, simplified acute physiology score; MV, mechanical ventilation; PCI, percutaneous coronary intervention; CABG, coronary artery bypass grafting.

**TABLE 5:** Cox regression analyses of risk factors for in-hospital mortality among coronary care unit patients with AMI (after PSM).

Variable	Univariate analysis		Multivariate analysis	
	HR (95% CI)	*P*	HR (95% CI)	*P*
Age	1.008(0.991–1.026)	0.366		
Gender (male)	1.184(0.804–1.744)	0.392		
CCI	0.891(0.809–0.982)	0.019	0.851(0.768–0.943)	0.002
LODS	1.048(0.997–1.102)	0.066	0.941(0.852–1.039)	0.228
OASIS	1.009(0.989–1.029)	0.398		
SAPS II	1.018(1.004–1.031)	0.010	1.020(1.002–1.040)	0.017
SAPS III	1.012(1.005–1.019)	<0.001	1.014(1.001–1.026)	0.038
MV	0.687(0.470–1.005)	0.053	0.372(0.226–0.614)	<0.001
PCI/CABG	0.860(0.573–1.289)	0.465		
AKI	0.609(0.324–1.143)	0.122		
AG	1.120(1.067–1.177)	<0.001	1.105(1.049–1.064)	<0.001

PSM, propensity score matching; AMI, acute myocardial infarction; HR, hazard ratio; CI, confidence interval; CCI, charlson comorbidity index; AG, anion gap; AKI, acute kidney injury; LODS, logistic organ dysfunction system; OASIS, oxford acute severity of illness score; SAPS, simplified acute physiology score; MV, mechanical ventilation; PCI, percutaneous coronary intervention; CABG, coronary artery bypass grafting.

### Comparison of receiver operating characteristic curves

**FIGURE 2:**
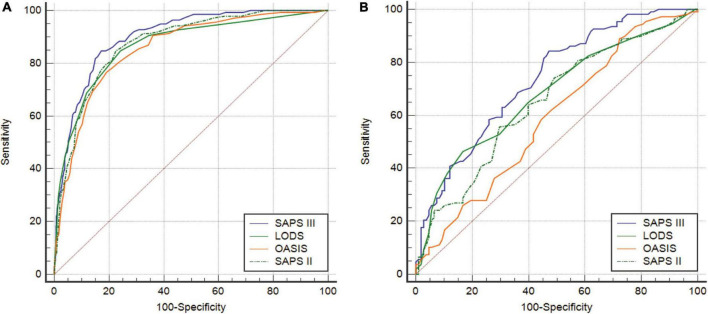
**(A)** ROC curves of LODS, OASIS, SAPS II, and SAPS III before PSM. **(B)** ROC curves of LODS, OASIS, SAPS II, and SAPS III after PSM. ROC, receiver operating characteristic; LODS, logistic organ dysfunction system; OASIS, oxford acute severity of illness score; SAPS, simplified acute physiology score; PSM, propensity score matching.

The ROC curves were used to demonstrate the predictive value of the four scoring systems for in-hospital mortality in CCU patients with AMI. All AUCs for the four scoring systems were greater than 0.850 before PSM. The AUC of the SAPS III score was greater than that of LODS, OASIS, and SAPS II (Figure 2A). Although LODS, SAPS II, and SAPS III had the same sensitivity, SAPS III had the highest specificity (82.83%) and Youden’s index (0.6750) (Table 6). After PSM, the AUC of the SAPS III model was 0.736, which was superior to LODS (*Z* = 2.541, *P* = 0.011), OASIS (*Z* = 4.960, *P* < 0.001), and SAPS II (*Z* = 3.219, *P* = 0.004) in predicting in-hospital mortality (Figure 2B). As shown in Table 7, SAPS III demonstrated reasonable sensitivity (84.26%) and Youden’s index (0.3704).

**TABLE 6:** Comparison of ROC curves (before PSM).

Factor	AUC	95%CI	Optimal cut-off	Sensitivity	Specificity	Youden’s index	*P*-value	*Z* value
LODS	0.868	0.846–0.887	4	84.67	75.86	0.6053	0.0045	2.838
OASIS	0.856	0.834–0.877	34	76.64	80.85	0.5750	0.0016	3.149
SAPS II	0.876	0.855–0.895	37	84.67	77.52	0.6219	0.0415	2.039
SAPS III	0.901	0.882–0.918	49	84.67	82.83	0.6750	Ref	Ref

ROC, receiver operating characteristic; PSM, propensity score matching; AUC, area under the ROC curve; LODS, logistic organ dysfunction system; OASIS, oxford acute severity of illness score; SAPS, simplified acute physiology score; CI, confidence interval; *P*-value, compared with SAPS III.

**TABLE 7:** Comparison of ROC curves (after PSM).

Factor	AUC	95%CI	Optimal cut-off	Sensitivity	Specificity	Youden’s index	*P*-value	*Z* value
LODS	0.680	0.613–0.742	8	46.30	83.33	0.5547	0.011	2.541
OASIS	0.591	0.522–0.657	28	88.89	27.78	0.1667	<0.001	4.960
SAPS II	0.652	0.584–0.715	47	55.56	70.37	0.2593	0.004	3.219
SAPS III	0.736	0.672–0.0.794	49	84.26	52.78	0.3704	Ref	Ref

ROC, receiver operating characteristic; PSM, propensity score matching; AUC, area under the ROC curve; LODS, logistic organ dysfunction system; OASIS, oxford acute severity of illness score; SAPS, simplified acute physiology score; CI, confidence interval; P-value, compared with SAPS III.

### Kaplan–Meier curves of the simplified acute physiology score III scoring system

**FIGURE 3:**
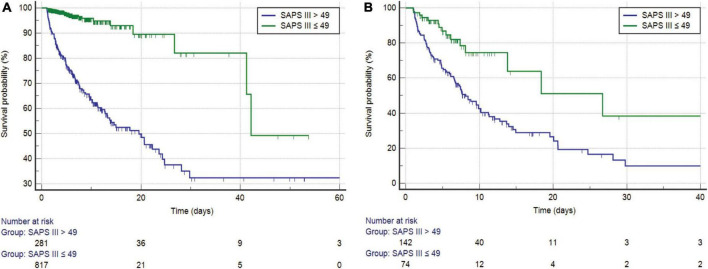
**(A)** Kaplan–Meier survival curves of SAPS III for the study cohort before PSM (log-rank *P* < 0.0001). **(B)** Kaplan–Meier survival curves of SAPS III for the study cohort after PSM (log-rank *P* < 0.0001). SAPS, simplified acute physiology score; PSM, propensity score matching.

Before PSM, SAPS III was separated into high- and low-score categories, with an optimal cutoff of 49 (Figure 3A). The Kaplan–Meier curves showed a median survival of 19.566 days for the high-score group (95% CI: 13.185–24.301) and 42.156 days in the low-score group (95% CI: 41.237–42.156). The difference between the two groups was statistically significant (log-rank test, *P* < 0.0001), and the hazard ratio (HR) of the high-score group was 7.636 (95% CI: 5.355–10.889) when compared with the low-score group. After PSM, the optimal cutoff value was 49 (Figure 3B). The median survival days in the high-score group were 15.245 days (95% CI: 10.795–19.695), whereas they were 24.493 days in the low-score group (95% CI: 16.694–32.292). The difference between the two groups was statistically significant (log-rank test, *P* < 0.0001). Moreover, the HR for the high-score group was 2.077 (95% CI: 1.379–3.123) compared to the low-score group.

### Comparison of decision curve analysis curves

**FIGURE 4:**
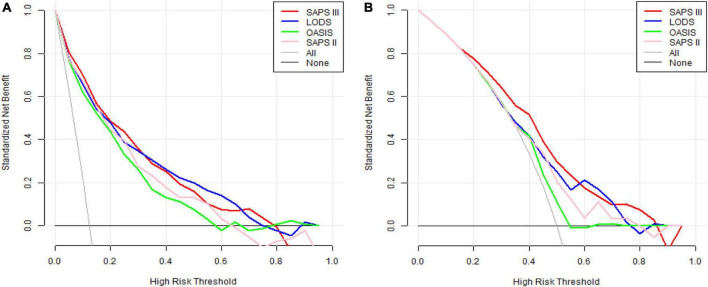
**(A)** DCA curves of LODS, OASIS, SAPS II, and SAPS III before PSM. **(B)** DCA curves of LODS, OASIS, SAPS II, and SAPS III after PSM. DCA, decision curve analysis; LODS, logistic organ dysfunction system; OASIS, oxford acute severity of illness score; SAPS, simplified acute physiology score; PSM, propensity score matching.

Before PSM, the DCA curve results of the four scoring systems showed that SAPS III and LODS had slightly more clinical profits than OASIS and SAPS II, but the two overlapped and were comparable (Figure 4A). After PSM, SAPS III had the greatest net benefit when compared to the other three scoring systems if the high-risk threshold was lower than 0.6, while the net benefit of LODS was slightly higher than SAPS III when the high-risk threshold was between 0.6 and 0.7. When the high-risk threshold was greater than 0.8, SAPS III offered more significant net benefits than the other three scoring systems (Figure 4B).

## Discussion

This study explored a better scoring system to predict in-hospital mortality in CCU patients with AMI. The main findings are as follows: (1) The death group had higher mean LODS, OASIS, SAPS II, and SAPS III scores than the survivor group; (2) SAPS III was an independent risk factor for predicting in-hospital mortality in CCU patients with AMI before and after PSM; (3) SAPS III had a more excellent predictive value than the other three scoring systems; (4) before and after PSM, there was a substantial increase in in-hospital mortality and a significant decrease in median survival days as the SAPS III score increased; (5) SAPS III provided the greatest net benefits across the largest threshold range compared to the other three scoring systems; and (6) patients who underwent PCI/CABG and those who had coexisting comorbidities were not associated with in-hospital mortality.

We analyzed the independent risk factors associated with AMI by binomial logistic regression and cox regression analysis. Regression analysis revealed that SAPS III was the independent predictor of mortality in CCU patients with AMI before and after PSM. AKI is a severe complication of AMI, and the development of AKI is strongly correlated with in-hospital mortality (18, 19). Before PSM, approximately 92.0% of patients in the death group died from AKI-related comorbidities, which was significantly higher than in the survival group. To make the data from the death and survival groups more comparable, we matched the two groups. Thus, the baseline characteristics of the patients in each group were comparable to reduce the influence of potential confounding factors. AG was included due to the limited number of variables and its high odds ratio value in the univariable regression analysis. The results suggested that AG provided significant predictive value for in-hospital mortality in CCU patients with AMI. Indeed, previous studies demonstrated that AG was an independent and reliable predictor of all-cause mortality after AMI. Higher AG values were associated with more severe clinical types of coronary artery disease (20, 21). More clinical data collected in the public MIMIC-IV database will increase the credibility of the analysis. Mortality in individuals with AMI is related to rapid percutaneous coronary intervention (PCI), mechanical problems, and indications of heart failure (22–24). Patients with STEMI who underwent primary PCI had a 10-year survival rate of 76.2% after hospital discharge (25). PCI/CABG seemed like an independent risk factor for in-hospital mortality of patients with STEMI (26). However, the conclusion is inconsistent with the current results. One possible explanation is that the surgical population in our study had comparable baseline characteristics before and after PSM.

It is crucial to determine the risk level in critically ill patients. The prevalence of major diseases and changes in diagnosis and treatment will lead to poor discrimination and calibration of scoring systems. Depending on the individual conditions and geographic regions, the performance of scoring systems differs in various scenarios (27). Critical care clinicians are seeking a scoring prognostic model that is standardized, highly precise, and specific. In a retrospective study, the common variables of LODS, including Glasgow Coma Scale (GCS) score, heart rate, blood pressure, urine output, and PaO_2_/FiO_2_, were mostly related to STEMI outcomes. Therefore, LODS could better predict in-hospital mortality in ICU patients with STEMI (26). Our study found that SAPS III has the second highest sensitivity and Youden’s index. The AUC of the LODS model was 0.680, which was lower than the 0.736 of SAPS III after PSM (*P* = 0.011, *Z* = 2.541). Consequently, SAPS III has greater discriminative power than LODS. Furthermore, the OASIS system had fewer variables and displayed higher sensitivity than other scoring systems, but it had the lowest specificity. In a cohort study of clinical outcomes in patients with sepsis, OASIS might be an initial predictor, as it does not contain any laboratory parameters (28). Some laboratory parameters, such as platelet count, white blood cell, and serum creatinine level, have specific values for diagnosing AMI and predicting mortality after AMI (29–31). Due to the lack of these meaningful laboratory parameters, OASIS is not an ideal predictor of patients with AMI. The capacity of SAPS III to differentiate in-hospital mortality was superior to that of the other three scoring systems. This may be because the biggest advantage of SAPS III compared to other databases is that it provides both global and regional databases for users to choose from according to the situation. To more accurately predict mortality in different geographic regions, the SAPS III scoring system uses a different calculation formula for each region (32). Special and appropriate criteria can be selected for ICU patients to predict mortality. Another feature of SAPS III, the manual collection and calibration of data within 1 h of ICU admission, prevents mortality prediction from being interfered with by clinical interventions or increased sampling rates (33). Related research compared the performance of the SAPS II and SAPS III in intermediate-care patients, and the SAPS II had adequate discriminative power but was poorly calibrated and might underestimate patient mortality (34). Consequently, the SAPS III showed high accuracy in predicting mortality.

SAPS III demonstrated excellent discrimination for 28-day mortality in individuals with sepsis (35). Furthermore, SAPS III was more sensitive and discriminative than the Acute Physiology and Chronic Health Evaluation (APACHE) IV scoring system for mortality prediction in multi-trauma ICU patients (36). Another study with 1042 ICU patients came to the same conclusion: The SAPS III score was more predictive of mortality than the APACHE II score (37). To date, no studies have evaluated the predictive power of the SAPS III scoring system for in-hospital mortality in patients with AMI. The present results confirmed that SAPS III was an independent risk factor for predicting in-hospital mortality in CCU patients with AMI. Meanwhile, SAPS III had a significantly higher AUC than LODS, OASIS, and SAPS II. This may be because SAPS III contains hydrogen ion concentration, which is not included in the other three rating systems. Hydrogen ion concentration was confirmed to be related to myocardial ischemia and reperfusion injury (38). A stratification analysis of the optimal cutoff for SAPS III showed that higher scores were associated with higher in-hospital mortality.

Decision curve analysis is one method to evaluate the clinical utility of predictive models. We observed the DCA curves of all scoring systems. Before PSM, the DCA curves of SAPS III and LODS had marginally greater clinical profits than OASIS and SAPS II, but this advantage was not significant. After PSM, in most threshold ranges, the clinical benefit of SAPS III was significantly higher than that of the other three scoring systems. However, it was only slightly lower than that of LODS within a very minimal range of about 0.6–0.7. This suggested that when most thresholds were selected for mortality prediction in clinical applications, the SAPS III score appeared to provide patients with the greatest clinical benefit. For example, if we use 0.8 as the threshold value, doctors will take active measures to reduce death as much as possible in clinical practice when patients have an 80% probability of death. Meanwhile, SAPS III’s ordinate (red line) is roughly 0.08, but the horizontal coordinates of the other three scores were less than or equal to 0. This meant that if the SAPS III score had been used to predict death at this threshold, eight out of every hundred patients would have benefited clinically, whereas the other three scoring systems would not have. The interpretation of thresholds in other locations is the same. Therefore, SAPS III is an excellent model for predicting in-hospital mortality for CCU patients with AMI.

As a strength of the study, two groups were defined by the optimal cutoff for SAPS III, and a subgroup analysis was performed. Before and after PSM, the risk of death remained higher in the high-score group than in the low-score group, which added to the reliability of the results. It is critical to consider the SAPS III score in clinical practice. Early identification and prompt intervention in patients with AMI at high risk for mortality can help reduce in-hospital mortality. Another strength is the extensive and effective data from MIMIC-IV. A large-scale investigation was conducted to increase the credibility of the study. There are also some limitations to this study. First, this was a single-center study based on a predominantly white US population, which may have potential implications for the analysis. Second, longitudinal data generated during hospitalization, such as dynamic changes in the scoring systems, may also be useful for prognostic prediction. However, we were unable to normalize these aforementioned longitudinal data due to the large variation in the length of hospital/CCU stay among patients; thus, only the scoring system scores on the first day of admission were selected for this study. Factors influencing mortality after CCU discharge were not considered, which became increasingly pertinent over time. Third, some key markers associated with myocardial injuries, such as troponin T or troponin I, were not compared with scoring systems in the study. That is because complete troponin values were unavailable in hospitalized patients, and blood troponin levels fluctuate rapidly, making it difficult to determine the exact value at the onset of a disease, which had an impact on outcomes. Therefore, prospective, large-scale clinical studies can be performed to validate the results.

## Conclusion

SAPS III is an independent risk factor for CCU patients with AMI. The predictive value for in-hospital mortality with SAPS III is superior to that of LODS, OASIS, and SAPS II. The results of the DCA analysis suggest that SAPS III may provide a better clinical benefit for patients. We demonstrated that SAPS III is an excellent scoring system for predicting in-hospital mortality in CCU patients with AMI.

## Data availability statement

The original contributions presented in this study are included in the article/supplementary material, further inquiries can be directed to the corresponding author.

## Ethics statement

Ethical review and approval was not required for the study on human participants in accordance with the local legislation and institutional requirements. Written informed consent for participation was not required for this study in accordance with the national legislation and the institutional requirements.

## Author contributions

XZ and TH contributed to the study design and drafted and revised the manuscript. XZ contributed to the design and acquisition of data. TL contributed to the statistical analysis. WW and TH contributed to the design and critically revised the manuscript. All authors contributed to the article and approved the submitted version.

## Footnotes

1
https://mimic.mit.edu/

2
https://physionet.org/content/mimiciv/1.0/
